# Single-Step Fabrication of Highly Tunable Blazed Gratings Using Triangular-Shaped Femtosecond Laser Pulses

**DOI:** 10.3390/mi15060711

**Published:** 2024-05-28

**Authors:** Jorge Fantova, Ainara Rodríguez, Luis Omeñaca, Oihane Beldarrain, Gemma G. Mandayo, Santiago M. Olaizola, José Lens, Mikel Gomez-Aranzadi

**Affiliations:** 1 Ceit-Basque Research and Technology Alliance (BRTA), Manuel Lardizabal 15, 20018 Donostia/San Sebastián, Spain; 2 Departamento de Ingeniería Eléctrica y Electrónica, Universidad de Navarra, Tecnun, Manuel Lardizabal 13, 20018 Donostia/San Sebastián, Spain; 3 Laintec, A.I.E. (Daisalux), Ibarredi 4, 01015 Vitoria-Gasteiz, Spain

**Keywords:** ultrafast laser, pulsed laser ablation, beam shaping, surface texture, micro-fabrication, groove morphology

## Abstract

Blazed gratings are periodic surface structures of great interest for applications such as friction control, light trapping, and spectrometry. While different laser processing methods have been explored to produce these elements, they have not yet surpassed conventional surface manufacturing techniques, often based on lithography processes or mechanical ruling. This work introduces a new approach based on the combination of ultrashort pulses and triangular beam shaping, which enables the generation of asymmetrical grooves in a single step. The main advantage of this strategy is that by simply changing the laser processing direction we can induce a significant modification in the ratio of asymmetry between the sidewall angles of the machined channels. The paper includes a comprehensive study, which has been supported by statistical tools, of the effect of this and other experimental parameters on the morphology of grooves machined on stainless steel. As a result, we achieved a wide range of geometries, with asymmetry ratios spanning from 1 to 5 and channel depths between 3 and 15 µm. Furthermore, we demonstrate the validity of the approach through the successful manufacture of blazed gratings of various slopes. The results reflect the versatility and cost-efficiency of the proposed fabrication strategy, and thus its potential to streamline the production of sawtooth gratings and other devices that are based on asymmetrical features.

## 1. Introduction

In the field of surface materials processing at the micrometer scale, the generation of asymmetric groove features is of great interest in fields such as wavelength filtering [1] and friction control [2,3]. When arranged in a periodic fashion, they can form sawtooth-shaped gratings, also known as echelle or blazed gratings, which are elements capable of collecting incoming light and selectively diffracting it toward a specific order [4,5]. These devices can be tuned for their use in different fields based on their topographic parameters, such as the duty cycle or the asymmetric tilt angle of the grooves, known as the blaze angle. Some applications of this technology include light trapping [6,7], X-ray reflecting gratings [8,9], spectrometry [10,11], and image projection [12,13].

The fabrication of blazed gratings has traditionally relied on mechanical machining [14,15], lithographic processes [16,17], or anisotropic wet etching [18,19] to reproduce the target designs. Due to their high operating costs, both time- and resource-wise, these methods are typically employed to manufacture either a mask or a master, which is then used to transfer the pattern onto the substrate of interest. Common replication methods involve nanoimprint lithography [7,20], hot embossing [11,21], or injection molding [17,22].

In recent years, ultrafast direct laser writing (DLW) has become a popular and cost-effective method for facilitating the fabrication process [23,24]. Several strategies have explored the inclusion of this technique, for example, 3D laser-assisted lithography, where electron beam lithography is substituted in the exposure of the photoresist [25,26], or oblique engraving, which can be achieved by significantly tilting the substrate with respect to the incident laser beam [27,28]. However, these approaches are not ideal because they either require a multi-step manufacturing process or involve operating in non-orthogonal positioning, which could make their industrial implementation challenging. To overcome such limitations, many authors have turned to beam shaping techniques, as they can often improve the throughput, precision, or versatility of current fabrication processes [29,30,31].

In view of this, the present work pushes toward the one-step fabrication of blazed gratings through the combination of ultrafast DLW and a triangular beam shaper. This diffractive optical element was used to transform the conventional Gaussian pulse distribution into a triangular-shaped flat-top profile. By manipulating the pulse overlap direction, we achieve the machining of asymmetrical grooves with varying sidewall angles on stainless steel substrates. Complementary, the adjustment of other experimental parameters, such as the processing speed or the number of laser passes, further refines the microstructuring process and broadens the range of potential groove geometries. Optical profilometry was employed to characterize the morphology of the channels, with the data subsequently analyzed with the aid of statistical tools. Following this parametric study, we explored the viability of the fabrication approach to produce blazed gratings.

## 2. Materials and Methods

### 2.1. Ultrafast Pulse Laser Processing Setup

**Figure 1:**
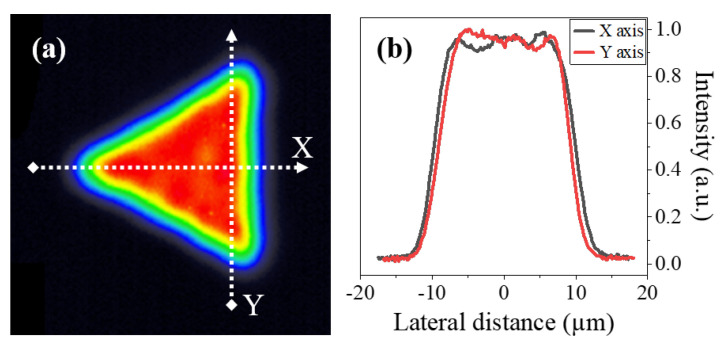
(**a**) Imaging of the Triangular flat-top beam used in the laser machining experiments. The white lines depicted in the image correspond to the movement directions of the X and Y linear stages.The false color contrast represents the intensity distribution of the beam. (**b**) Intensity profiles along the X and Y axes of the generated beam, as defined in (**a**).

The laser source used in this work was a *Satsuma HP* (Amplitude, Inc., San Francisco, CA, USA),which emits 280 fs pulses at 1030 nm with an output beam diameter of 2.5 mm and a quality of M<1.1. The beam was first narrowed to a diameter of 1 mm to fit into the triangular beam shaper, which was developed by Cailabs (Rennes, France). After passing through the shaping module, the triangular flat-top (TFT) profile was formed with a side length of 0.5 mm. The beam was then relayed by two consecutive telescopes with magnification factors of 1× and 25×, respectively, to reach the processing plane. The last lens in the system was a *MicroSpot* (Thorlabs Inc., Newton, NJ, USA) microscope objective with a 0.4 numerical aperture and a focal length of 10 mm, which effectively focused the beam down to a spot with a full width at half maximum of approximately 20 µm. An image of the resulting beam at the processing plane is shown in Figure 1a,b, where we can appreciate the homogeneity of the flat-top distribution and its triangular shape. The picture was recorded using a *LaserCamHR* (Coherent, Inc., Santa Clara, CA, USA) beam profiling camera.

The laser pulses were deposited using *ATS100* (Aerotech, Inc.,Pittsburgh, PA, USA) motorized linear stages, which allowed for movement in three axes, namely: X, Y, and Z. The relative orientation between the TFT beam and the positive translation of the axes is shown in Figure 1a. The polarization of the beam was parallel to the Y-axis throughout all the experiments. The substrates used for machining were 0.5 mm thick polished plates of AISI 304 stainless steel. This material was selected due to its widespread use in the industry and laser processing [32,33], in applications such as steel-tape gratings for optical encoders [34,35].

### 2.2. Writing Strategy

Unlike a circular shape, a triangular arrangement introduces asymmetry at certain planes of intersection, which means that the overlap pattern of TFT pulses can vary with the beam’s rotation. During the raster scanning of a line, each unique orientation of the beam would result in a different distribution of the deposited energy. This, in turn, could lead to the engraving of V-shaped channels of various tilts.

To study this approach, we conducted a simulation in Python that measured the energy deposited following the overlapping of TFT pulses along a raster line. We ran this process for four different rotation angles of the triangular shape, namely: 0, 30, 45, and 90°. We based the calculations on the intensity profile of the TFT beam shown in Figure 1. A more comprehensive description of the simulation process can be found in Appendix A.

**Figure 2:**
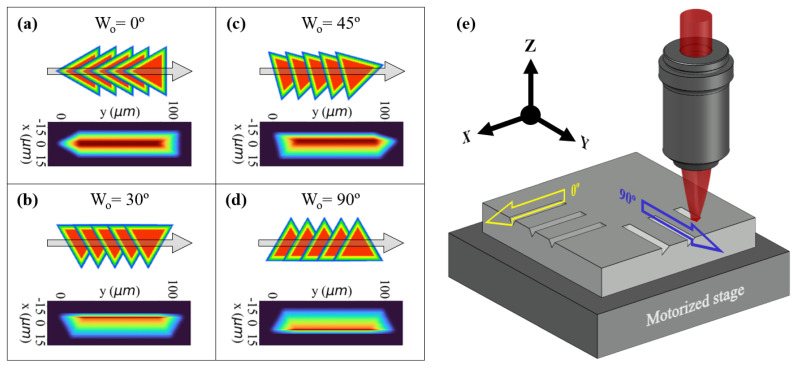
(**a**–**d**) Diagram illustrating the overlap of the triangular beam pulses along the scanning direction for each of the different writing orientations tested (top), and simulation of the energy exposure distribution associated with said overlap patterns along the scanning path (bottom). (**e**) Visual representation of the engraving strategy for the 0° and 90° writing orientations.

The results of the simulations are shown in Figure 2a–d. At the top of each figure section, one can see a diagram depicting the relative orientation between the processing direction and the TFT rotation angle, in addition to the resulting overlap of the pulses. At the bottom, we observe the output of the simulation, which shows the aforementioned dependence between energy deposition and rotation of the triangular shape. Thus, the calculations predict that machining with a ‘0°’ orientation of the TFT would result in a symmetric groove, similar to one that a Gaussian beam could produce. Meanwhile, the use of 30, 45, and 90° oriented beams would produce channels with a varying degree of asymmetry.

To simplify the optomechanical setup, we maintained the orientation of the beam shown in Figure 1a and, instead, we changed the angle of motion of the translation stage, i.e., the processing direction, to achieve the same energy deposition patterns depicted in Figure 2a–d. We configured the X and Y stages to move in a linear fashion at four distinct angles: 0, 30, 45, and 90°, which we will refer to hereafter as *writing orientation* ($W_{o}$). A $W_{o}$ of ‘0°’ corresponds to the exclusive translation of the X stage while the pulse deposition is taking place. Conversely, a $W_{o}$ of 90° entails that the Y stage is the only one in motion during the laser machining. Intermediate writing angles, such as 30 and 45°, involve the concurrent movement of both axes, with the ratio between their translation speed determining said angles. Figure 2e illustrates the proposed scanning strategy for the 0° and 90° writing orientations.

In addition to the writing orientation, we studied the influence of three other experimental parameters in the morphology of the grooves: pulse energy ($E_{p}$), the number of laser passes over the same groove (*N*), and the processing speed (*S*). The values of each parameter used in the experiments can be seen in Table 1. A total of 180 unique grooves were engraved using all possible permutations of the listed parameters. Lastly, the laser repetition rate (*f*) was set to 10 kHz for all the experiments.

### 2.3. Morphological Characterization

**Figure 3:**
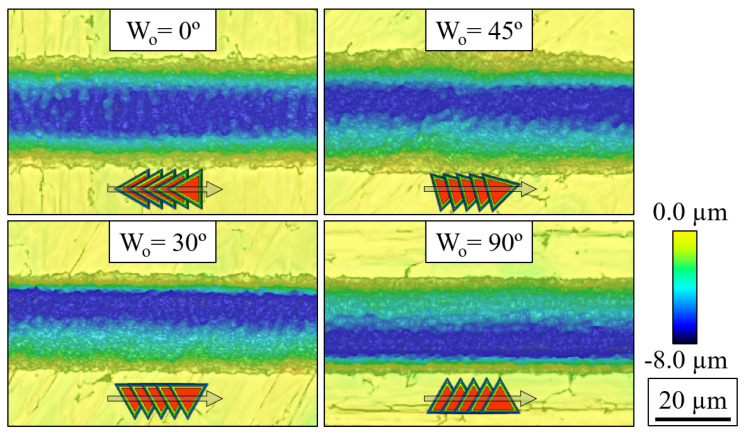
Example of the surface analysis provided by the optical profilometer for four grooves fabricated at different writing orientations, where the color contrast shows the height variation over the scanned region. The black scale bar applies to all pictures. The diagrams embedded in the images represent the corresponding overlap of the pulses during the machining of each of the channels.

Surface topography analysis of the fabricated grooves was performed using a Sensofar S-neox optical profilometer, which allowed for the three-dimensional mapping of the channels. We can observe some examples of the output of this process in Figure 3, which show the topography of four grooves with an average depth of ≈6 µm fabricated at different writing orientations.

**Figure 4:**
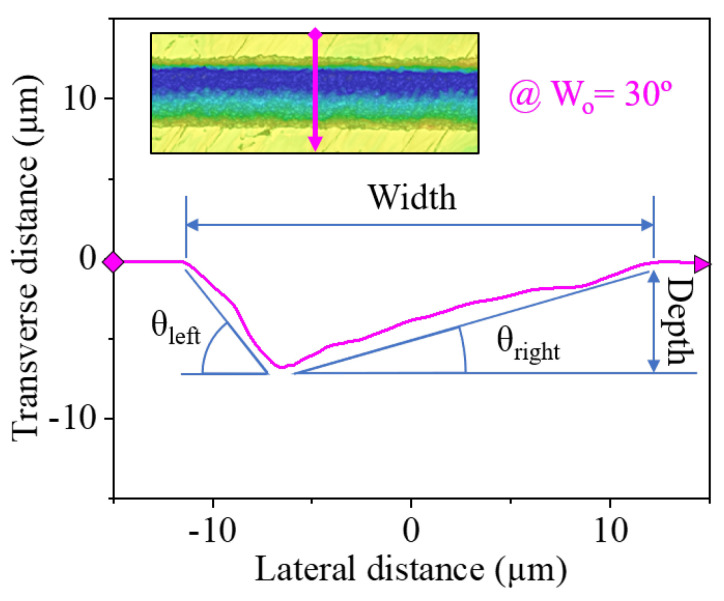
Transverse profile of a groove processed at a $W_{o}$ of $30^{\circ }$, which corresponds to the pink line highlighted in the inset image. The graph includes the line annotations used as a reference when measuring the four morphological parameters.

After such an analysis, we measured four distinct dimensions, henceforth referred to as *response variables*, for each of the fabricated grooves: ablated depth, the width of the trench, and tilt angles of the ‘left’ ($\theta_{left}$) and ‘right’ ($\theta_{right}$) sidewalls of the groove. To do this, we calculated the average cross-sectional profile across each channel, which resulted in a graph such as that of Figure 4. We then extracted the value of these response variables, using the line annotations shown in the aforementioned sample graph as reference points.

### 2.4. Statistical Analysis

**Figure 5:**
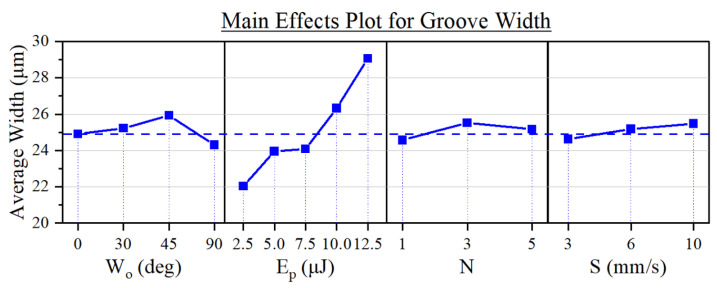
Main effects of the experimental parameters on the width of the grooves. The horizontal dashed line represents the average width among the 180 specimens.

Given the amount of specimens produced, we employed the Minitab 21 statistical software to analyze the data obtained from the morphological characterization. One of the main tools employed was the ‘*Main Effects Plot*’, which graphs the average of a response variable for each discrete value of the corresponding input parameter. The value of each point corresponds to the average among all the samples that were produced using said input parameter value. For instance, the ordinate value of ‘0°’ in the ‘$W_{o}$’ plot of Figure 5 is the result of averaging the width of all the grooves that were machined using a $W_{o}$ of 0°, which accounts for 45 grooves out of the total 180.

The experimental data of each response variable was also analyzed using the ‘*General Full Factorial Design*’ Minitab model. This enabled the identification of relevant ‘*interactions*’ between the input parameters, which stands for cases in which the combination of said input parameters holds a compound effect on a response variable, beyond the individual contribution of each parameter.

## 3. Results and Discussion

### 3.1. Influence of Experimental Parameters

After introducing all the experimental data extracted from the topography measurements, the statistical analysis revealed several findings regarding the effect of the processing parameters (Table 1) on the dimensions of the grooves. We discuss the influence of each input parameter in the following sections.

#### 3.1.1. On the Width of the Grooves

The width of the grooves proved to be the most constant response variable, holding an average value of 24.86 ± 2.76 µm among the 180 specimens. This is reflected in its Main Effects Plot, shown in Figure 5, where the variation in width was generally small compared to the said average. Pulse energy was found to be the most influential parameter.

**Figure 6:**
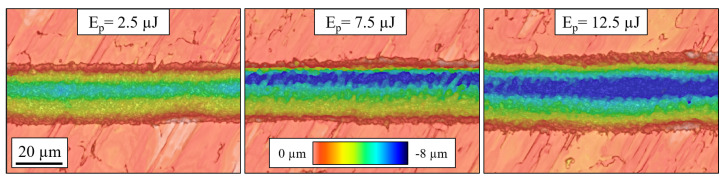
Optical profilometer micrographs of grooves fabricated at an increasing pulse energy. Common processing parameters: $W_{o}$ = 30º; *N* = 3; *S* = 6 mm/s.

Both of these phenomena can be attributed to the beam’s flat-top profile. On one hand, the combination of the steepness of the profile and the flatness of the beam at its peak (Figure 1b) allows for a more precise definition of the grooves [36,37] and, thus, less variability in the resulting width of the channels. On the other hand, the width of the beam profile varies slightly along the intensity of the pulse. For example, at normalized intensity values of 90, 50, and 10 %, the nominal width of the beam as per Figure 1b was 15.2, 19.7, and 24.2 µm, respectively. Thus, as the pulse energy increases, so does the area of the pulse that exceeds the ablation threshold of the material. This resulted in the expansion of the grooves seen in Figure 5: from a width of 22 µm when using 2.5 µJ pulses to around 29 µm with 12.5 µJ pulses. We can also observe this trend in the micrographs of Figure 6, where the widths of the channels were 23.7, 24.5, and 30.7 for $E_{p}$ of 2.5, 7.5, and 12.5 µJ, respectively.

#### 3.1.2. On the Depth of the Grooves

**Figure 7:**
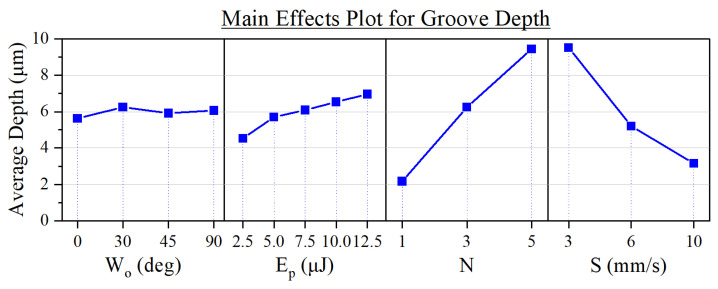
Main effects plot of the experimental parameters on the depth of the grooves.

Apart from the writing orientation, all input parameters affected the groove depth, as shown in Figure 7. Within the studied experimental range (Table 1), we were able to machine grooves as deep as 18.18 µm when using the following input parameters: $W_{o}$ = 30°; $E_{p}$ = 12.5 µJ; *N* = 5; *S* = 3 mm/s.

**Figure 8:**
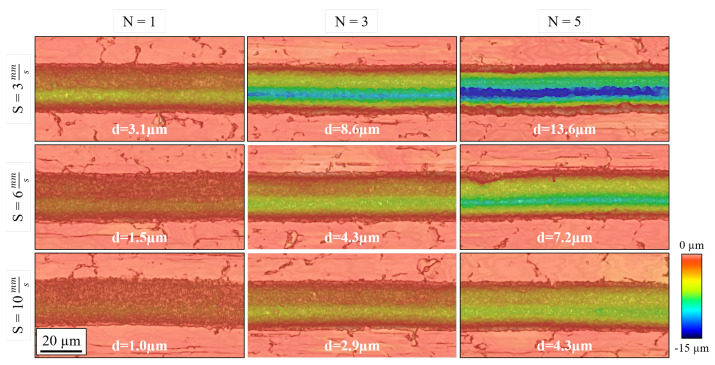
Optical profilometer micrographs of grooves fabricated at different *N* and *S* values. The peak average depth of each channel (d) is indicated. Common processing parameters: $W_{o}$ = 90°; $E_{p}$ = 2.5 µJ.

The number of laser passes and the processing speed were the most influential factors, probably because their variation entails a large shift in the number of deposited pulses throughout the engraving. We can see an example of their impact in the micrographs of Figure 8.

To assess the efficiency of the ablation process, we calculated the resulting specific removal rate, i.e., $\dot{V}/P_{av}$, for each experiment, which represents the ablated volume per unit of energy. Equation (1) describes this new response variable, adapted from [38] to fit our writing strategy. Specifically, we considered that the volume of the ablated groove is defined by its depth (*d*), width (*w*), and scanning length (*L*). Additionally, we assumed that the total number of pulses (*n*) is evenly deposited along this *L* distance, given the laser repetition rate (*f*) and processing speed (*S*). Finally, we also factored in the number of laser passes (*N*) performed in each experiment.

(1) $$\frac{\dot{V}}{P_{av}}=\frac{V}{n\cdot E_{p}}=\frac{d\cdot w\cdot L}{\left(N\cdot \frac{f}{S}\cdot L\right)\cdot E_{p}}=\frac{d\cdot w\cdot S}{N\cdot f\cdot E_{p}}\left[\frac{\mu m^{3}}{\mu J}\right]$$

**Figure 9:**
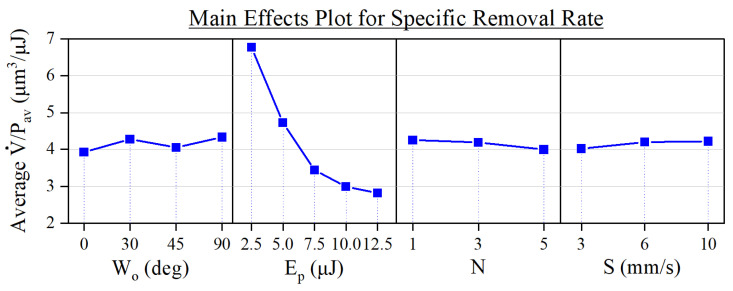
Main effects plot of the experimental parameters on the specific removal rate.

Following the calculation of the specific removal rate, we depict in Figure 9 the main effects plot for each of the involved input parameters.

On this occasion, we find that the influences of *N* and *S* are minimal compared to that of $E_{p}$. This contrasts with the trend that these parameters follow in Figure 7 and is likely caused by the normalization of the ablation volume to the number of deposited pulses performed in Equation (1). We also observe that the specific removal rate suffers a quadratic decrease as $E_{p}$ increases, making it the most relevant parameter regarding the efficiency of the ablation process. This tendency is similar to that reported by other authors for ablation processes performed with either Gaussian or flat-top beams on stainless steel [38,39], which suggests that a peak in specific removal rate may be found at pulse energies below 2.5 µJ.

**Figure 10:**
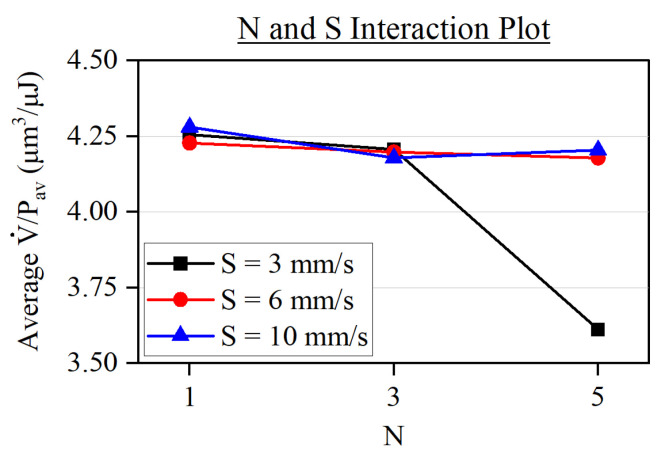
Interaction plot showing the compound effect of the number of laser passes and the processing speed on the specific removal rate.

As for the interactions between the input parameters, the most influential one for the specific removal rate was between *N* and *S*, whose associated graph can be found in Figure 10. There, we appreciate how the specific removal rate drops around 15% when selecting both a higher number of laser passes (N=5) and lower processing speeds (S=3 mm/s). Seeing as this combination also entails the machining of the deepest grooves, as shown in Figure 7, it is possible that this decrease in ablation efficiency could be linked to the occurrence of redeposition effects. This is a known factor that hinders the ablation process and one that is promoted the higher the accumulated fluence is [40,41].

#### 3.1.3. On the Tilt of the Grooves

**Figure 11:**
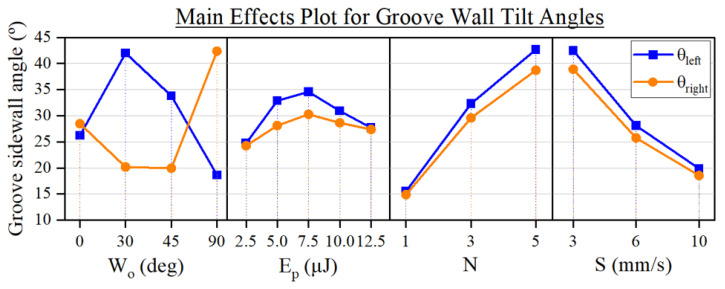
Main effects plot of the experimental parameters on the sidewall angles of the grooves, i.e., $\theta_{left}$ and $\theta_{right}$.

The slope of the sidewalls of the grooves was heavily influenced by all the input parameters, as can be seen in Figure 11. There, we observe how both sidewall angles ($\theta_{left}$ and $\theta_{right}$ as measured following Figure 4) exhibit almost a parallel trend regarding the effect of $E_{p}$, *N*, and *S*, whereas $W_{o}$ impacts each tilt angle in a distinct manner. By evaluating the ratio between the resulting sidewall angles, defined as $\theta_{R}=\theta_{left}/\theta_{right}$, we see that by changing $W_{o}$ we produce, on average, grooves of varying inclination: symmetric grooves ($\theta_{R}\approx 1$) for a $W_{o}$ of 0°, ‘left-tilted’ grooves ($\theta_{R}\approx 2$) for a $W_{o}$ of 30° and ‘right-tilted’ grooves ($\theta_{R}\approx 0.5$) for a $W_{o}$ of 90°. This confirms our expectations of the effect of the rotation of the TFT that we describe in Figure 2 since, for instance, the grooves produced at $W_{o}$ of 30 and 90° are effectively symmetrical to each other, which can also be appreciated in Figure 3.

As for the influence of *N* and *S* in the wall angles, we can point out that their impact is similar to the one they induced in the depth of the grooves (Figure 7). This can be explained by taking into account that these parameters did not have a notable effect on the width of the grooves (Figure 5). In other words, since the shift of these parameters produces a variation in the depth of the grooves but not in their width, the resulting $\theta_{left}$ and $\theta_{right}$ thus become dependent on the depth modulation, as determined by trigonometry.

**Figure 12:**
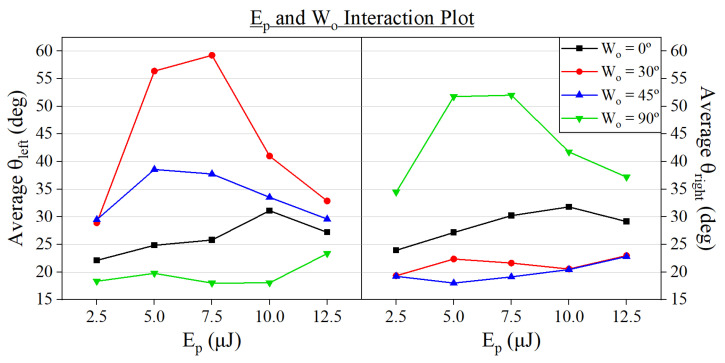
Interaction plot showing the compound effect of pulse energy and writing orientation on $\theta_{left}$ and $\theta_{right}$.

Regarding the interactions amid the input parameters, the most significant one identified for both $\theta_{left}$ and $\theta_{right}$ was between $E_{p}$ and $W_{o}$. Figure 12 shows the effect of both parameters on each wall angle. Given the value of the response variables at each writing orientation, we can extract distinct trends between two groups of grooves: the symmetric ($W_{o}=0$°) and the asymmetric ones ($W_{o}=30,45,90$°).

When producing asymmetric channels, the highest tilt angles were achieved on average at ‘intermediate’ pulse energies such as 5 µJ ($\theta_{left}=56.40$° with a $W_{o}$ of 30° and $\theta_{right}=51.77$° for a $W_{o}$ of 90°) and 7.5 µJ ($\theta_{left}=59.25$° with a $W_{o}$ of 30° and $\theta_{right}=52.02$° for a $W_{o}$ of 90°). Then, the use of higher $E_{p}$, such as 10 and 12.5 µJ, caused a decrease in the inclination of the channels. The appearance of this ‘sweet spot’ of high tilt angles could be attributed to the change in the width of the grooves since it greatly increases for pulse energies beyond 7.5 µJ as we saw in Figure 5. Due to how the angles are measured (Figure 4), this broadening of the grooves lowers both $\theta_{left}$ and $\theta_{right}$.

By analyzing Figure 12, we can determine that this phenomenon is enhanced the more asymmetric the groove is. We find that the shift in tilt angle caused by the increase of pulse energy from 2.5 to 7.5 µJ is greater in the case of machining at $W_{o}$ of 30°, where $\theta_{left}$ varies between 28.92 and 59.25°, and 90°, with $\theta_{right}$ ranging from 34.48 to 52.02° than it is at a $W_{o}$ of 45°, where $\theta_{left}$ changes between 29.49 and 38.58°. When the pulse energy rises over 7.5 µJ, the reverse occurs, as the tilt angles decrease at a more rapid rate for $W_{o}$ of 30 and 90° than for a $W_{o}$ of 45°.

Regarding the machining at $W_{o}=0$°, both angles steadily increased at a similar rate along pulse energy until reaching a maximum at 10 µJ, where $\theta_{left}\approx \theta_{right}=31.5$°, after which they suffered a slight reduction.

### 3.2. Versatility of the Fabrication Approach to Control the Geometry of the Grooves

**Figure 13:**
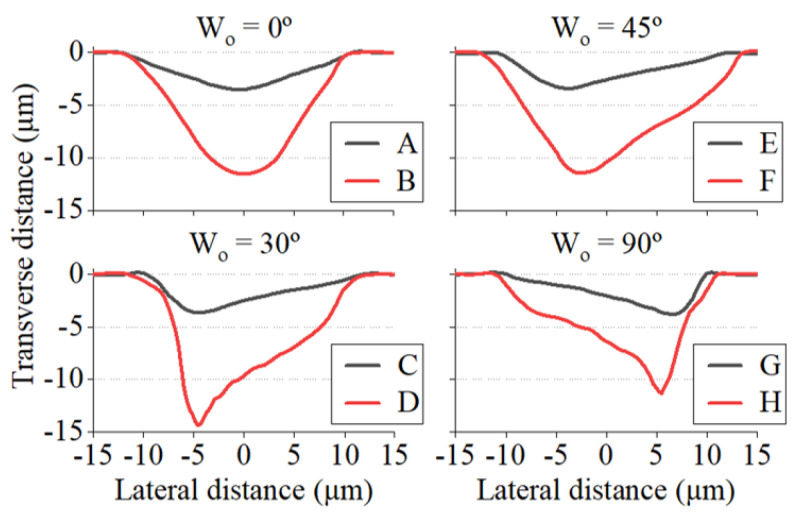
Transverse profiles of the grooves listed in Table 2.

After reviewing the influence of each of the input parameters, we were able to generate grooves of very diverse geometries. Among these, eight specimens particularly stood out due to their significant variations in sidewall angle ratios at groove depths ranging from 3 to 15 µm. Their morphological parameters are detailed in Table 2, while their transverse profile can be found in Figure 13.

Examining both the table and the figure, we can confirm once again that the writing orientation plays a critical role in the tilting of the grooves. In this regard, the *D* and *G* grooves stand out: the former due to its high $\theta_{left}$ of nearly 80°, and the latter due to its high right-tilt asymmetry of $\theta_{R}$ = 0.2. In the context of blazed grating fabrication, the blaze angle of the grooves typically refers to the sidewall with the lowest tilt [18,42]. Therefore, it would be 40.7 and 13.1° for the D and G grooves, respectively.

It is worth noting that higher asymmetry ratios were predominantly observed in channels with a depth of 5 µm or lower. However, this ratio decreased on average as the depth of the grooves increased, as reflected by the samples listed in Table 2.

### 3.3. Fabrication of Blazed Gratings

To test the viability of the fabrication strategy to produce blazed gratings, we proceeded to machine parallel grooves at a periodicity in the order of the width of the TFT beam. We aimed toward the production of gratings of high asymmetry and low depth, as it results in lower blaze angles, a much sought-after feature for applications such as X-ray reflective gratings [9,20].

**Figure 14:**
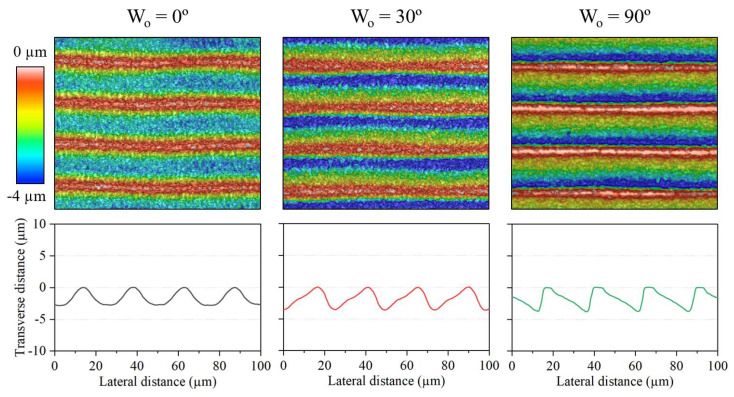
Optical profilometry micrographs of blazed gratings fabricated at different writing orientations (**top**) and their corresponding height profile (**bottom**). Common processing parameters: $E_{p}$ = 5.0 µJ; *N* = 1; *S* = 3 mm/s.

Based on the discussion of the last sections regarding $\theta_{R}$ and, in particular, the results shown in Table 2, we selected the following experimental parameters to promote the aforementioned geometry of the gratings: $E_{p}$ = 5.0 µJ; *N* = 1; *S* = 3 mm/s. Under these conditions, we engraved the grooves at a $W_{o}$= 0, 30, and 90° while maintaining a period of 24.5 µm. Figure 14 shows the topography and transverse profile of the resulting microstructures.

We can observe that the influence of $W_{o}$ on the groove asymmetry is carried over when fabricating the gratings. The resulting $\theta_{R}$ of the gratings written at 0, 30, and 90º were of, approximately: 1.0, 2.7, and 0.2. Meanwhile, their blaze angle was around 20.6º, 13.5º, and 12.0º, respectively. These values are in the range of those displayed in the individual grooves when engraving under the same conditions, i.e., those of A, C, and G of Table 2.

The height profile of Figure 14 also reveals the homogeneity of the gratings over the scanned region. However, we note that the machining resulted in a duty cycle of over 100% for the first two gratings, and approximately 90 % for the third one. This estimation is based on the presence or absence of a flat plateau at the *Transverse distance* = 0 mark, which corresponds to the unprocessed steel substrate. Therefore, lateral beam overlap must have occurred during the laser engraving of the first two gratings. The fact that this did not occur for $W_{o}$ = 90° could be linked to the higher mechanical stability of the Y stage with regard to the X stage since it is the only one moving at this $W_{o}$.

## 4. Conclusions

In this work, we established that the combination of triangular beam shaping with femtosecond lasers enables the straightforward and versatile fabrication of blazed gratings on stainless steel in a single step.

We successfully engraved asymmetrical grooves with various geometries by adjusting the laser processing parameters. The most influential factor proved to be the relationship between the orientation of the triangular beam and the direction of the pulse overlap. The impact of other variables, such as pulse energy or processing speed, was aligned with our expectations. Nonetheless, the flat-top distribution of the beam proved key to keeping the width of the grooves almost constant for most of the engravings performed, thus contributing to the stability of the process. As a result of the extensive parametric study, we attained sidewall tilt angles as high as ≈80° and ratios of asymmetry of up to 5. Lastly, we validated the manufacturing approach by generating homogeneous sawtooth gratings with blaze angles of 12º on the surface of steel substrates.

The reported results show that this fabrication strategy can produce asymmetric V-shaped channels in a flexible and efficient manner. Therefore, triangular beam shaping represents a promising alternative to the conventional manufacture of blazed gratings, riblet surfaces for aerodynamic purposes, and other devices that rely on asymmetric microstructures, especially when considering the integration of state-of-the-art beam delivery systems to enhance the throughput of the process. 

## Figures and Tables

**Table 1:** Range of processing parameters used in the study.

Writing Orientation, $W_{o}$ [°]	Pulse Energy, $E_{p}$ [µJ]	Number of Laser Passes, *N*	Processing Speed, *S* [mm/s]
0; 30; 45; 90	2.5; 5.0; 7.5; 10; 12.5	1; 3; 5	3; 6; 10

**Table 2:** Summary of the experimental and morphological parameters of eight distinct grooves.

Condition	Depth [µm]	$\theta_{R}$	$\theta_{\mathrm{left}}$ [°]	$\theta_{\mathrm{right}}$ [°]	Width [µm]	$W_{o}$ [°]	$E_{p}$ [µJ]	*N*	*S* [mm/s]
A	3.6	1.0	19.0	19.3	24.6	0	5.0	1	3.0
B	11.5	0.9	52.8	56.7	24.9	0	2.5	5	3.0
C	3.8	3.2	41.2	12.9	23.4	30	5.0	1	3.0
D	14.4	1.9	79.0	40.7	24.8	30	7.5	5	3.0
E	3.3	2.6	31.5	12.3	23.4	45	5.0	1	3.0
F	11.9	1.4	53.9	38.2	26.9	45	10.0	3	3.0
G	3.9	0.2	13.1	57.7	21.5	90	5.0	1	3.0
H	11.3	0.5	34.7	64.6	23.3	90	7.5	3	3.0

**Figure A1:**
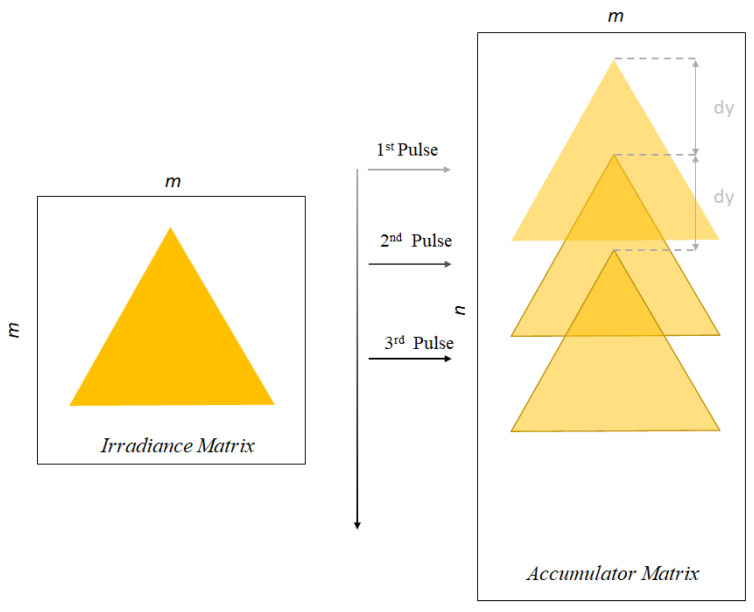
Schematic of the tessellation and accumulator method. The disposition of the TFT beam overlap corresponds to its ‘0º’ rotation state.

## Data Availability

The raw data supporting the conclusions of this article will be made available by the authors upon request.

## Appendix A. Modeling the Exposure of Triangular Flat-Top Beam Pulses

To understand how the triangular flat-top (TFT) beam shape shown in Figure 1 will affect the laser ablation process, it is crucial to anticipate the energy distribution profile produced by the overlap of the laser pulses. Our initial hypothesis is that the slope of the sidewalls of the laser-machined grooves is influenced by the relationship between the processing direction and the orientation of the TFT beam.

To study this, we simulated the overlap of TFT pulses along a set distance at a fixed scanning direction, i.e., the y-axis, while rotating the TFT beam shape at different angles, namely: 0, 30, 45, and 90°. To take into account different overlap values, we used the ratio between the laser frequency, i.e., the repetition rate, and the processing speed to determine the distances between pulses, dy, as per Equation (A1).

(A1) $$dy=\frac{\mathrm{scan speed}(\frac{mm}{s})}{frequency(kHz)}$$

Knowing both this distance and the spot size, which can be extracted from Figure 1, the overlap between two pulses can be calculated following Equation (A2). For the simulation, we selected a repetition rate of 10 kHz and a scan speed of 2 mm/s, which corresponds to an overlap of 99%.

(A2) $$Overlap\left(\%\right)=\frac{\mathrm{spot size}-dy}{\mathrm{spot size}}$$

In addition to the overlap and TFT rotation angle, we also considered the total number of pulses in the simulation. These parameters allowed us to use the tessellation and accumulator scheme to calculate the distribution of energy density. Figure A1 shows a schematic example of how this method works.

This technique is intended for modeling non-uniform or non-symmetric laser beams. An *m × m* irradiance matrix was created from the real image of the top hat obtained by a beam profiler. The size of each pixel was used to rescale the axes of the matrix to obtain the real size. An accumulator matrix *m × n*(n≫m) was also created and initialized to zero. After the first pulse, the first *m × m* elements of the accumulator matrix were updated with the values from the irradiance matrix. The contribution of the second pulse involved updating the values of the accumulator by adding the values from the irradiance matrix, considering the shift dy. Using this summation method and the corresponding offset iteratively, the accumulated energy density in the material can be modeled for arbitrary angles, pulse numbers, and overlap values.

The output of the model can be seen in Figure 2, where the effect of the rotation of the TFT beam shape drastically affects the exposure distribution.
